# Investigation on the antimicrobial activity of chitosan-modified zinc oxide-eugenol cement

**DOI:** 10.1080/26415275.2019.1697621

**Published:** 2019-12-12

**Authors:** Inger Sofie Dragland, Hanne Wellendorf, Hilde Kopperud, Ida Stenhagen, Håkon Valen

**Affiliations:** Nordic Institute of Dental Materials, Oslo, Norway

**Keywords:** Chitosan, zinc oxide, eugenol, antibacterial

## Abstract

**Introduction:** Coronal leakage and reinfection after root canal therapy is an important reason for endodontic failure. Zinc oxide-eugenol (ZOE) -based materials are often used as a coronal seal to prevent secondary infection. The antibacterial effect of ZOE cement is mainly due to leaching of eugenol from the material, but the effect is reported to decrease over time. Chitosan (CH) is a natural polymer with antibacterial properties. The aim of the study was to investigate if incorporation of (CH) and chitosan oligosaccharide (COS) in a ZOE-based material improved both the immediate and sustained antibacterial properties of the material.

**Methods:** *Enterococcus faecalis*, *Streptococcus mutans* and *Staphylococcus epidermidis* was used to investigate the antibacterial effect of the materials in a modified direct contact test (MDCT) immediately after setting and after storage for 18 weeks in water. Leaching per week of eugenol from the materials was quantified using gas chromatography–mass spectrometry (GC-MS). The effect of eugenol on growth of bacteria was measured by reading of optical density at 600 nm after 18 h growth. Mechanical properties were investigated in a compressive strength test according to ISO 3107.

**Results:** The present study showed that a ZOE-based material has antibacterial activity both as freshly prepared and after immersion in water for 18 weeks. Incorporating CH or COS may increase the antibacterial effect depending on the bacterial species investigated. The amount of leached eugenol did not differ between materials or during or after storage. *S. mutans* showed the highest susceptibility to eugenol of the three species investigated. Modification of the materials with CH or COS reduced the compressive strength, but the requirements in ISO 3017 were still met.

## Introduction

Coronal leakage after root canal treatment is considered to be an important reason for failure of the endodontic treatment due to contamination of the root canal system [1]. *In vitro* studies have shown that rapid penetration of bacteria of the entire root canal system may occur after endodontic treatment without a coronal seal over the root filling [2,3]. The presence of a base coronal to the root filling has been shown to reduce microleakage [4] and increase the long term prognosis of root canal-treated teeth [5]. However, microbial leakage has been shown to occur even after placement of temporary filling materials coronal to the root filling [6]. This shows that the material’s used for a coronal seal, should have the ability to seal the orifice of the root filling and possess antibacterial properties. The ability of materials to prevent bacterial leakage have, however, been clinically shown to differ between materials [7]

A zinc oxide-eugenol (ZOE)-based restorative material is one of the most commonly used temporary restorative materials in dentistry. In endodontics, a ZOE restorative material is also used as a base under the permanent restoration to prevent access of bacteria to the root canal both between appointments and after the permanent restoration is placed [8]. The antibacterial and bacteriostatic effects of ZOE-based materials are partly mediated by leaching of eugenol. The free hydroxyl groups in eugenol are thought to cause damage to the cell membrane by altering the permeability of the membrane followed by leakage of the cellular contents [9]. Other proposed antibacterial mechanisms are the production of intracellular reactive oxygen species (ROS), inhibition of membrane bound adenosine triphosphatase (ATPase) enzymes activity involved in ion transportation and adenosine triphosphate (ATP) generation [9–11]. The antibacterial effect depends on both contact time and type of bacteria [12,13]. The antibacterial effect of ZOE-based restorative materials has been reported to decrease over time [13]. When ZOE materials are exposed to water or saliva, eugenol will diffuse slowly out of the material due to hydrolysis of zinc eugenolate [14].

Chitosan (CH) is a natural, biodegradable, bioactive, and non-toxic compound produced by deacetylation of chitin, found in the exoskeleton of crustaceans. Chitosan oligosaccharide (COS) is produced by enzymatic degradation or acidic hydrolysis of chitosan or chitin. Chitosan is soluble in dilute aqueous acids (pH < 6.1), while COS is soluble in water [15]. Chitosan and COS exert an antimicrobial effect against bacteria that is dependent on the degree of acetylation and the molecular weight of the chitosan polymer [16–18]. The antibacterial effect is thought to be mediated by interaction of the cationic NH_3_^+^ groups in chitosan with the negatively charged bacterial cell wall, leading to increased membrane permeability and cell lysis [19].

Chitosan has been investigated in a wide range of dental applications such as materials for periodontal tissue regeneration, oral drug delivery, caries prevention, and incorporation in dental restorative materials [20]. Chitosan incorporation in bioglass, has been shown to increase remineralisation of enamel whit spot lesions by acting as a vehicle for the released ions from the bioglass [21]. When used as a coating on polystyrene, dental implants and poly(methylmethacrylate) surfaces, chitosan has been shown to reduce biofilm formation [22–24]

High diversity of microbes in both primary and secondary endodontic infections is reported [25,26]. The antibacterial effect of ZOE materials incorporated with CH or COS was assessed using three different bacterial species. *Enterococcus faecalis* and *Streptococcus mutans* were used as model organisms for bacteria identified in secondary root canal infections. *Staphylococcus epidermidis*, a human skin commensal, is an example of bacteria that may be transferred to the pulpal chamber and root canal during treatment and which have been identified in clinical cases with persistent symptoms [27,28]. In, addition the presence of bacteria on materials used for endodontic treatments such as rubber dam, have been shown [29].

The aims of the present study were to: (i) incorporate low molecular weight CH and COS into a ZOE-based restorative material to investigate the immediate and sustained antibacterial effect of the modified materials; (ii) investigate the amount of eugenol leaching from the modified materials and the antibacterial effect of eugenol on bacteria; (iii) evaluate mechanical properties of the materials after modification.

## Materials and methods

### Microorganisms

Stock cultures of *Staphylococcus epidermidis* (ATCC 35984), *Streptococcus mutans* (ATCC 700610) and *Enterococcus faecalis* (ATCC 29212) for experimental use were prepared from −70 °C culture in Brain Heart Infusion medium (BHI) (Oxoid Ltd, Basingstoke, UK) and grown over night at 37 °C and 5% CO_2_.

For use in the modified direct contact test (MDCT), the overnight culture was centrifuged for 5 min at 5000 g and re-suspended in phosphate-buffered saline (PBS, Lonza, Walkersville, USA) to a cell density of approximately 1 × 10^8^ colony forming units (CFU)/ml. For the measurement of optical density (OD) and growth rate after 18 h, overnight culture of bacteria was diluted 1/100 in media with eugenol (0–1250 μg/ml).

### Materials

Low molecular weight chitosan (CH) (89% deacetylated, 50–190 kDa) and chitosan oligosaccharide (COS) (5000 kDa) were purchased from Sigma-Aldrich (448869 and 523,682 St. Louis, USA). Intermediate Restorative Material (IRM) Ivory Standard Package (Dentsply, York, USA) was used as Zinc oxide-eugenol (ZOE) material. Zinc oxide powder was mixed with COS and CH powder respectively in a ratio of 20 wt% before the addition of eugenol (250 μl/g powder), and mixed to achieve a mouldable paste. The amount of chitosan incorporated was chosen based on previous results from incorporation in methacrylate based composite and bonding materials [30]. The material was left to set overnight in a moist environment at 37 °C.

### Modified direct contact test (MDCT)

For the MDCT of fresh material, a thin layer of the material was coated on the side walls of a 48-well microtiter plate. Test discs for investigating MDCT after 18 weeks were immersed in sterile Milli-Q water, and stored at 37 °C with regular replacement of water.

An amount of 10 µl of bacterial suspension (ca. 10^6^ bacteria) was placed on the surfaces of the material and as a control on polystyrene surfaces of the microtiter plate. The plates were kept at 37 °C (*S.mutans* in 5% CO_2_) for 1 h to allow the bacteria to come in direct contact with the material. Phosphate buffered saline (PBS) (1 ml) was added to the wells and serial diluted before 2 × 50 µl drops from each sample was plated on BHI agar and incubated overnight at 37 °C and 5% CO_2_. CFU were counted on the following day. At least 4 parallels from three separate experiments were performed for each of the different materials and controls. Live bacteria was expressed as numbers of CFU.

### Live dead staining of *S. epidermidis* after MDCT

A LIVE/DEAD™ *Bac*Light™ Bacterial Viability Kit, for microscopy (Thermo Fisher Scientific Waltham, USA) was used to confirm results from the MDCT on ZOE-based material with and without CH in a fluorescence microscope (Olympus Fluoview FV1200MPE, Tokyo, Japan).

### Quantification of eugenol

Material test discs for studying leaching of eugenol were made using teflon rings (*d* = 8 mm, *h* = 2.8 mm). Test discs were immersed in sterile Milli-Q water (3 ml/disc) and kept at 37 °C. Every week for 18 weeks the water was changed and the amount of eugenol was analysed in the aliquot removed after week 1, 4 and 18.

Gas chromatography–mass spectrometry (GC-MS) was used to perform quantification of eugenol in the aliquots [Agilent Technologies 780 A GC System-5975 Series- with a mass selective detector, model number 63170 A with a HP 5MS UI column installed (Agilent J&W GC Columns, Santa Barbara, CA., USA)].

Five, individually prepared, samples of eugenol of known concentration (Sigma Chemical Co., St. Louis, MI, USA) , together with an internal standard were analysed at 150 °C for 6 min. The MS Scan parameters were set to 40–600 *m/z*. Linear regression was used to establish the calibration curve in the range (6–90) µg/ml. The calibration curve had a correlation coefficient of 0.998 without forcing zero. The MS limit of quantification of eugenol was 0.1 µg/ml.

### Effect of eugenol on bacterial growth

Bacteria were grown in 96-well plates in BHI with different concentrations of eugenol (0–1250 μg/ml). Optical density at 600 nm was measured in a Multi-Detection Microplate Reader (Synergy H1, BioTek, Winooski, USA) after 18 h.

### Compressive strength

Specimens with a diameter of 4 mm and height of 6 mm respectively for compressive strength were made in moulds as described in ISO standard 3107:2011 (Dentistry – Zinc oxide/eugenol and zinc oxide/non-eugenol cements) [31]. The specimens were first kept in water at 37 °C for 24 h and then in water at 23 °C for 15 min before testing. Compressive strength was performed with a cross head speed of 0.75 mm/min using the Lloyd LRX testing machine (Lloyd Instruments Ltd, Hampshire, UK).

### Statistical analysis

The antibacterial assays was analysed using One-way ANOVA for comparison of the materials compared to control at each time point. The effect between the materials were analysed using Two-way ANOVA followed by Tukey’s multiple comparisons test for the simple effect of time and material. Eugenol release from the materials was analysed using Two-way ANOVA with repeated measures (mixed model ANOVA) followed by Tukey’s multiple comparisons test for the simple effect of time and material. The compressive strength values were log transformed and analysed using One-way ANOVA followed by the Tukey’s multiple comparisons test. A significance level of *p* < .05 was used for the analysis. The analyses were performed with GraphPad Prism version 7.01 for Windows (La Jolla, USA).

## Results

### Modified direct contact test (MDCT)

**Figure 1.**
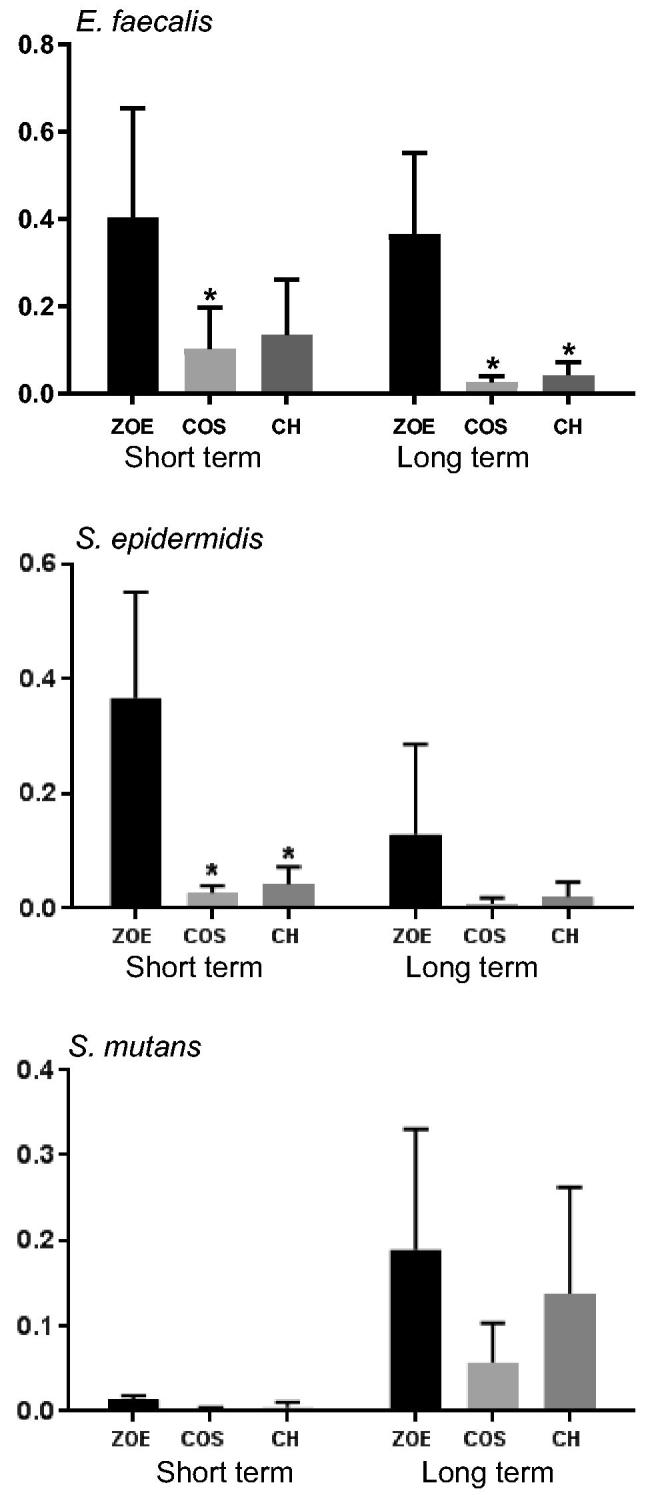
Number of CFU normalized to the control after 60 min MDCT on fresh material (Short term) and on material after 18 weeks in water (Long term) (*N* = 4). CH and COS (20%) labeled with * indicates significant statistical difference compared to ZOE.

**Figure 2.**
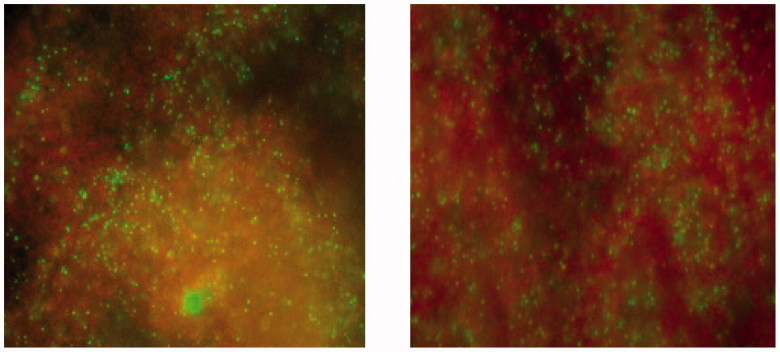
Live/dead staining of *S. epidermidis* after MDCT on unmodified ZOE (left) and ZOE with 20% CH (right). Green indicates live cells and red dead cells.

All materials both freshly prepared and after 18 weeks of storage in water, reduced the number of CFUs compared to control (Figure 1). For the analysis of the effect between the materials, there was a significant effect of the material factor for *E. faecalis* and *S. epidermidis*. For *E. faecalis* only the modified material with addition of 20% COS reduced the number of CFUs compared to the unmodified material for freshly prepared materials. After 18 weeks both modified materials reduced the number of CFUs compared to unmodified material. For *S. epidermidis* only fresh materials with either 20% COS or CH reduced the number of CFUs significantly compared to the unmodified material. For *S. mutans* there were no significant effect of the material factor. Live/dead staining of *S. epidermidis* after MDCT confirmed the results with higher proportion of red fluorescent cells, dead cells, on ZOE material containing CH compared to the unmodified ZOE-based material was observed (Figure 2).

### Quantification of eugenol leaching

**Figure 3.**
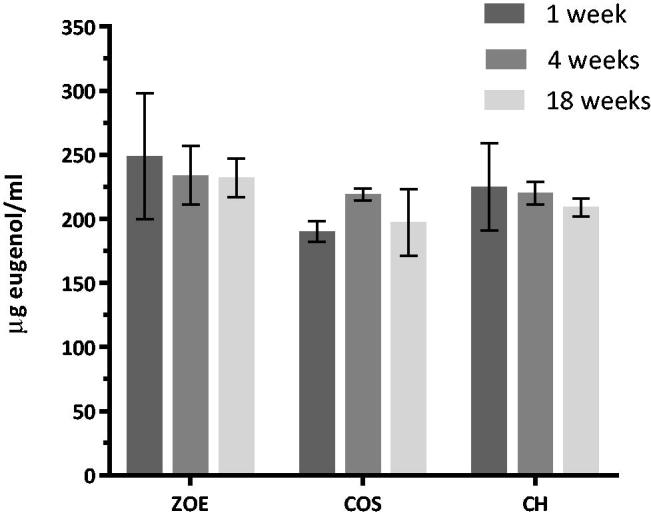
Amount of eugenol leaching from ZOE discs with and without CH or COS where the water was replaced every week and leaching during one week after 1, 4 and 18 weeks in water was analysed (*N* = 6).

The amount of eugenol leaching from the unmodified ZOE-based material and the CH and COS-modified materials was quantified to be in the range 200–250 μl/ml after one week in water (3 ml/disc). The analysis of the release of eugenol showed no significant effect of material or time. The multiple comparison test showed a significant reduced release of eugenol at week 1 for the 20% COS containing materials compared to the other materials, no other differences was observed (Figure 3).

### Effect of eugenol on bacterial growth

**Figure 4.**
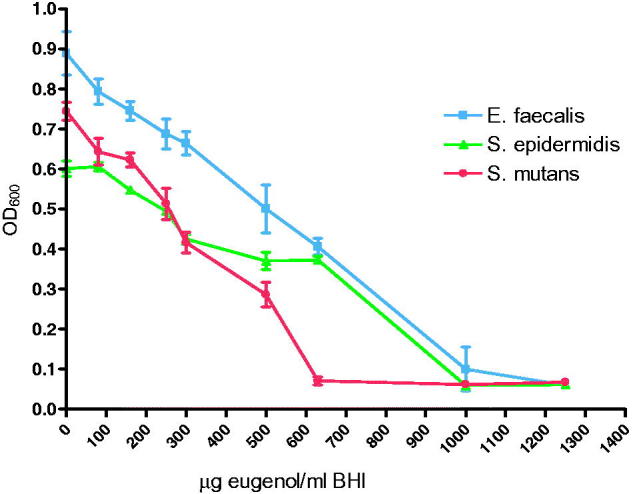
Growth curve of bacteria after 18 h measured at OD 600 nm in medium with eugenol (0–1250 μg/ml) (*n* = 6).

Eugenol showed a concentration dependent inhibition of growth for all bacteria investigated (Figure 4). The minimum inhibitory concentration (MIC) was lowest for *S. mutans,* while the highest MIC was observed for *E. faecalis* (Table 1).

**Table 1.** Minimum inhibitory concentration, MIC, after 18 h growth in medium with eugenol (µg/ml).

Bacterial strain	*E. faecalis*	*S. epidermidis*	*S. mutans*
MIC (µg/ml)	1200	1000	600

### Compressive strength

**Figure 5.**
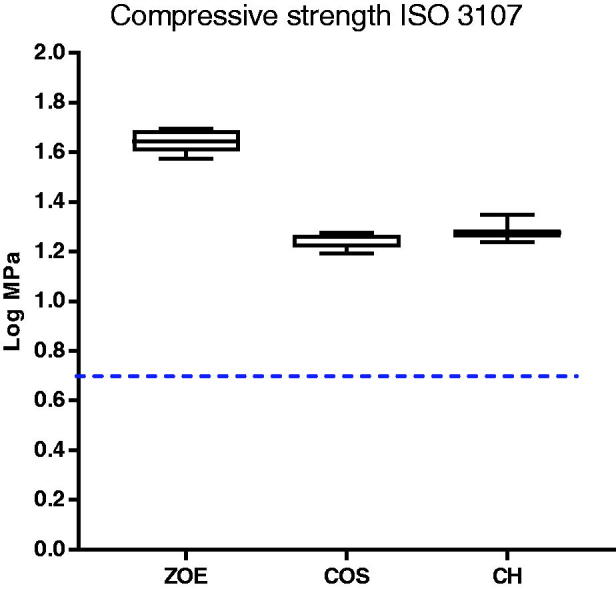
Compressive strength of ZOE specimens without and with 20% CH or COS as described in ISO 3107(values log transformed). The minimum requirement of 5 MPa is indicated by the horizontal dotted line (*N* = 5 for ZOE, *N* = 11 for COS and CH).

The compressive strength decreased significantly when the material was incorporated with CH and COS. However, the modified materials complied with the requirements of minimum 5 MPa, described in ISO standard 3107 for type II materials set for bases and temporary restorations (Figure 5). The test showed no significant difference in compressive strength between CH and COS.

## Discussion

A base coronal to the root filling with sustained antibacterial effect would aid in prevention of recontamination of the root canals between endodontic treatment sessions and after the permanent root filling. ZOE-based materials have been shown to possess antibacterial effects due to eugenol leaching out of the material [13,32–34]. However, the antibacterial effect against *S. mutans* and *E. faecalis* has been shown to be lost 14 and 30 days after setting, respectively [13]. The modified direct contact test (MDCT) used in this study showed that the ZOE-based unmodified material still showed an antibacterial effect after immersion in water for 18 weeks. The discrepancy with previously published studies [13,32] may be due to the difference in storage conditions for the test specimens, phosphate-buffered saline with and without antibiotic versus water, the bacterial strains,- the type of direct contact test (DCT) performed and the thickness, amount, of material used. The modified direct contact test performed in the present study investigates the bactericidal effect of the material after a certain time point, rather than the bacteriostatic effect investigated by the DCT [32,35].

Due to eugenol being released from temporary restorative materials over time [36], chitosan was incorporated in the material to increase and provide a sustained antibacterial effect [37]. Chitosan has previously been shown to possess an antibacterial activity when incorporated in a dental resin composite and when coated on dental implants [23,30,38]. Increasing the eugenol content may not be recommended due to its cytotoxic effect on human osteoblastic cells [39].

The antibacterial effect of chitosan is thought to be mediated by their positively charged amino groups, which may interact with the bacterial cell surface. The antibacterial activity has been reported to depend on the molecular weight of chitosan and the degree of deacetylation [18]. Different susceptibility of chitosan oligosaccharides towards *S. mutans* and *S. epidermidis* has previously been reported [40]. Compared to chitosan in solution where the bacteria are embedded and exposed to amino groups of chitosan, the bacteria in the MDCT test are exposed to chitosan on the surface of the material. The release of COS or CH from the materials could contribute to the antibacterial effect. While COS is soluble in water, CH is insoluble in water but soluble in acidic solutions with pH <6.1, such as acetic acid and lactic acid. The possibility of chitosan, mainly COS, solving into water from the modified materiel could over time be a parameter to take into consideration and to investigate further. In the present study, the bacteria was applied to the material in a 10 µl drop that evaporates, so the release of COS and CH on the antibacterial effect is most likely small. In addition, for the antibacterial activity there were no effect of time for *S. epidermidis* or *E. faecalis*, implying that little COS or CH is released from the material surface during immersion in water.

For *S. mutans*, no difference in susceptibility was observed between the unmodified ZOE-based material and the modified materials containing CH or COS. However, *S. mutans* showed increased susceptibility to eugenol compared to both S*. epidermidis* and *E. faecalis* as shown by the MIC values in the present study. This finding may explain why the effect of COS or CH was not apparent in the MDCT assays. In addition, different susceptibility of chitosan oligosaccharides towards *S. mutans* and *S. epidermidis* has previously been reported. In the MDCT, with test specimens with a volume of 0.14 cm^3^ in 3 ml water, the leaching of eugenol was measured to be in the range of 200–250 μg/ml. From the 18 h growth curve of eugenol, reduced growth of all three bacteria at this eugenol concentration was observed. The growth curve showed different effects of eugenol on the bacteria investigated with the largest effect on *S. mutans* (MIC 600 μg/ml), followed by *S. epidermidis* (MIC 1000 μg/ml), and *E. faecalis* (MIC 1200 μg/ml). With the exception of *S. mutans*, where an earlier study has shown MIC of eugenol as low as 100 μg/ml [10], results reported for *S. epidermidis* and *E. faecalis* are similar to the results reported in this study [41,42]. However, on the material surface the concentration of eugenol may be higher than the eugenol concentration measured in water/liquid and therefore differ from the concentration affecting the bacteria in the MDCT test, where bacteria are in direct contact with the material surface. The growth curve of *S. mutans* showed a high susceptibility to eugenol. This could explain the MDCT results for *S. mutans* where no difference between the modified and unmodified materials regarding the antibacterial effect was observed.

Reduced antibacterial activity of ZOE-based materials over time has been previously reported [34] and thought to be caused by reduced release of eugenol from the materials over time. However, leaching of eugenol has previously been shown to be stable for 33 days when the thickness of the specimens investigated was over 1.0 mm, and to diminish after up to approximately 20 weeks depending on the ZOE-based material [36,43]. In the present study, we replaced 20% of zinc oxide powder with CH or COS which could affect the amount of eugenol release from the material. To investigate this possible effect, the total amount of released eugenol per week was observed after 1, 4 and 18 weeks storage in water for the three different materials. These results showed that leaching of eugenol is a continuous and slow process and that the contribution of eugenol as an antibacterial agent was present in both MDCT tests on fresh material and on material after storage. These findings correspond with the antibacterial activity observed for the unmodified ZOE-based material.

Zinc oxide mixed together with eugenol will form an amorphous gel before setting to a cement of zinc oxide embedded in a matrix of zinceugenolate. Incorporating chitosan into this matrix may also influence the mechanical properties of the original material. After incorporating either CH or COS in the material, a decrease in the compressive strength was observed for both materials with no significant difference between them. However, the modified materials still met the requirements of minimum 5 MPa stipulated in ISO 3017. The standard sets a number of different requirements to ZOE materials. This is important to take into account in future work whit chitosan modified ZOE.

## Conclusion

The present study showed that a ZOE-based material has an antibacterial effect and that incorporating CH or COS may increase the antibacterial effect depending on the bacterial species. The total release of eugenol from the materials was stable for up to 18 weeks immersion in water. Eugenol therefore contributed to the antibacterial effect on all the material surfaces investigated. *S. mutans* exhibited the highest susceptibility to eugenol. The compressive strength of the modified materials was reduced but still met the requirements in ISO 3017.
